# The Neuroprotection of Verbascoside in Alzheimer’s Disease Mediated through Mitigation of Neuroinflammation via Blocking NF-κB-p65 Signaling

**DOI:** 10.3390/nu14071417

**Published:** 2022-03-29

**Authors:** Shanshan Chen, Honghan Liu, Shimiao Wang, Hongbo Jiang, Le Gao, Lu Wang, Lesheng Teng, Chunyue Wang, Di Wang

**Affiliations:** 1 School of Life Sciences, Jilin University, Changchun 130012, China; chenss20@mails.jlu.edu.cn (S.C.); honghan20@mails.jlu.edu.cn (H.L.); wangsm19@mails.jlu.edu.cn (S.W.); jianghb19@mails.jlu.edu.cn (H.J.); gaole1319@mails.jlu.edu.cn (L.G.); wanglu1319@mails.jlu.edu.cn (L.W.); tenglesheng@jlu.edu.cn (L.T.); 2 Engineering Research Center of Chinese Ministry of Education for Edible and Medicinal Fungi, School of Plant Protection, Jilin Agricultural University, Changchun 130118, China

**Keywords:** Alzheimer’s disease, verbascoside, neuroinflammation, neuroprotection, NF-κB signaling

## Abstract

Verbascoside (VB) is a phenylethanoid glycoside extracted from the herbaceous plant *Verbascum sinuatum* and plays a neuroprotective role in Alzheimer’s disease (AD). The goal of this study was to explore the neuroprotective mechanism of VB. Based on the proteomics analysis, immunohistochemistry, immunofluorescence, Western blot, and ELISA were utilized to explore the neuroprotective mechanism of VB in context of neuroinflammation in APP/PS1 mice, LPS-induced BV2 cells, and/or Aβ_1-42_-stimulated N2a cells. Proteomic analysis demonstrated that the neuroprotection of VB correlated closely to its anti-inflammatory effect. VB significantly blocked microglia and astrocyte against activation in brains of APP/PS1 mice, suppressed the generation of IL-1β as well as IL-6, and boosted that of IL-4, IL-10 and TGF-β in vivo, which were analogous to results acquired in vitro. Furthermore, VB effectively restrained the phosphorylation of IKKα+β, IκBα, and NF-κB-p65 in APP/PS1 mice; LPS-induced BV2 cells, and Aβ_1-42_-stimulated N2a cells and lowered the tendency of NF-κB-p65 translocation towards nucleus in vitro. These results demonstrate that the neuroprotective effect of VB correlates to the modulation of neuroinflammation via NF-κB-p65 pathway, making VB as a hopeful candidate drug for the prevention and treatment of AD.

## 1. Introduction

Alzheimer’s disease (AD) belongs to the spectrum of neurodegenerative diseases that occur mostly in elderly individuals aged over 65 years [1] with the clinical characteristics of memory lapse and cognitive impairment [2]. Extracellular amyloid-beta (Aβ) plaques that are formed because of the inappropriate digestion of amyloid precursor protein, as well as intracellular neurofibrillary tangles (NFTs) caused by the hyperphosphorylated tau protein, are generally accepted as pathological hallmarks [3]. Owing to an insidious onset, long latency, and various ambiguous mechanisms, it is difficult to explore an effective therapy for AD. Among the various hypotheses for pathological mechanisms of AD [4,5], neuroinflammation has been the current focus of research [6].

Neuroinflammation is the inflammation that occurs in the central nervous system (CNS) [7] and is physiologically a defense mechanism against endogenous waste and exogenous pathogens, thus promoting tissue repair and maintaining brain hemostasis [8]. However, continuous neuroinflammation triggers and aggravates neurodegeneration. Microglia, the innate immune cells resident in CNS, persistently monitor changes in the environment [9]. When the brain is subjected to various stimuli such as pathogens, the microglia execute defense functions, including synapse remodeling and removal of cellular debris, in response to these changes, thereby maintaining brain homeostasis [10]. Once the Aβ plaques outside neurons are recognized by the microglia, on the one hand, they are activated to release pro-inflammatory factors leading to neuroinflammation [11], and on the other, they migrate and gather around the plaques to engulf them [12]. Disruption of this balance between neuroinflammation and phagocytosis accelerates the progression of AD [13]. Nuclear factor (NF)-κB, a transcription factor, exists in almost every type of cells. It is involved in the regulation of inflammatory events [14], during which NF-κB signaling is activated to produce more pro-inflammatory factors, that is, tumor necrosis factor (TNF)-α, interleukin (IL)-1β, and IL-6. IL-1β and TNF-α further interact with the remaining microglia, astrocytes, and neurons to amplify the signals of neuroinflammation, thereby aggravating AD pathology. However, exogenous transforming growth factor (TGF)-β was verified to repress NF-κB signaling in Aβ-induced glial cells, thus attenuating inflammation [15].

Because of multiple pharmacological activities, natural products are being increasingly screened for agents to treat various diseases [16,17]. Sodium oligomannate, which is commercially named as GV-971, has been reported to treat mild-to-moderate AD by regulating neuroinflammation implicated by amino acids of the gut bacteria [18]. Verbascoside (VB), also called acteoside, is a phenylethanoid glycoside extracted from the herbaceous plant *Verbascum sinuatum* (Figure S1). VB was reported to inhibit the NOD-like receptor family protein 3-mediated acute inflammatory injury in an intracerebral hemorrhage mouse model [19]. Inhibition of acetylcholinesterase and β-secretase levels [20,21] and suppression of Aβ plaque accumulation in Aβ_1-42_-induced AD rats by VB [22] suggests its neuroprotective role in AD. In our previous study on (APPswe/PSEN1dE9)/Nju double transgenic male AD model mice (APP/PS1 mice), VB inhibited the formation of Aβ deposits and NFTs partly through inhibiting endoplasmic reticulum stress [23]. Although VB exhibits anti-inflammatory and neuroprotection particularly in AD, the molecular mechanism has not yet been clarified. 

Our team tended to further elucidate the neuroprotection of VB via its anti-neuroinflammatory activity in APP/PS1 mice, lipopolysaccharide (LPS)-induced BV2 cells, and Aβ_1-42_-stimulated N2a cells. This study focuses on how VB exerts neuroprotective function in AD from the molecular level, thereby paving the road for clinical use of VB as a treatment for the disease.

## 2. Materials and Methods

### 2.1. Animal Feeding and Agent Administration

Eight-month-old male B6C3-Tg APP/PS1 mice (genotype: [Appswe] T, [Psen1] T) and male wild type (WT) (genotype: [Appswe] W, [Psen1] W) were delivered by the Nanjing Biomedical Research Institute of Nanjing University. All mice were raised in a thermostatic room (humidity, 40–50%; temperature, 21–24 °C). In a 12 h light/dark cycle, free access to food and water were offered to all mice. APP/PS1 mice were stochastically assigned to two groups: (i) the model group that was orally treated with 0.4 mL of normal saline (*n* = 12) daily for 6 weeks and (ii) the VB-treated group that was orally treated during the same period with 10 mg/kg of VB (61276-17-3, 98.38% purity, Chengdu Herbpurify Co., Ltd., Chengdu, China), which was firstly dissolved with DMSO (1/2000 V_total_) before diluting with normal saline (*n* = 12) [23,24]. WT mice (*n* = 12) were administered 0.4 mL of normal saline daily for 6 weeks through the gastric route and served as the control group. All animal experimental procedures in this study were authorized by the Experimental Animal Center of Jilin University (Number: SY201905014) and ARRIVE guidelines. After a 6-week treatment, sodium pentobarbital (150 mg/kg) was given to mice for euthanasia. The blood and brain samples were collected thereafter. 

### 2.2. Label-Free Quantification Proteomics

Analogous to our previous study [23], 100 mg brain tissue samples were homogenized to collect supernatant, which was precipitated with acetone and incubated overnight at the temperature of 37 °C after adding trypsin and then desalted using a C18 column. Polypeptides acquired were subjected to LS-MS/MS analysis. The raw MS file was processed using MaxQuant (version 1.5.6.0), which obtained a database of protein sequences from UniProt (Uniprot_mouse_2016_09). The heat map and graph of protein–protein interactions were constructed using Origin 2022 (Northampton, MA, USA) software and the STRING website, respectively.

### 2.3. Cell Culture 

N2a cells (CX0020, Boster Biological Technology Co., Ltd., Wuhan, China), the mouse-derived neuroblastoma cells, were cultured with minimum essential medium (MEM) (PM150410) containing 10% fetal bovine serum (164210) and 1% penicillin-streptomycin solution (PB180120). BV2 cells (CL-0493), the mouse-derived microglia cells, were grown in BV2-specific medium (CM-0493). All reagents mentioned above and the BV2 cells were from Procell Life Science & Technology Co., Ltd. (Wuhan, China). 

For the co-culture, BV2 cells were subjected to the 3 h pretreatment with VB (50 μM and 100 μM), which was dissolved with DMSO (1/2000 V_total_) followed by dilution in basic MEM. Then, BV2 cells were subjected to 6 h incubation of 5 μM of Aβ_1-42_ (052487, Gill Biochemical Co., Ltd., Shanghai, China). The medium was replaced with fresh basic MEM, which was collected after 12 h and then given to N2a cells for 24 h before further testing. All cells were propagated at the condition of 37 °C and 5% CO_2_.

### 2.4. Cell Viability and Lactate Dehydrogenase (LDH) Cytotoxicity Assay

BV2 and N2a cells (5 × 10^3^ cells/well) were propagated in 96-well plates. BV2 cells were subjected to 3 h pretreatment of VB (50 μM and 100 μM) followed by 24 h stimulation with 1 μg/mL LPS (DH183-1, Beijing Dingguo Changsheng Biotechnology Co., Ltd., Beijing, China). N2a cells were subjected to 3 h pretreatment of VB (25 μM and 50 μM) followed by 24 h stimulation with 5 μM Aβ_1-42_. For cell viability assaying in the co-culture condition, N2a cells (5 × 10^3^ cells/well) were cultured in 96-well plates with BV2 culture medium at 100 μL/well, while for the LDH assay, N2a cells (2 × 10^4^ cells/well) were cultured in 24-well plates and stimulated using 1 mL BV2 culture medium per well. 

As in our previous study [23], the 3-(4,5-dimethyl-2-thiazolyl) 2,5-diphenyl-2H-tetrazolium bromide (MTT) (S19063, Shanghai Yuanye Bio-Technology Co., Ltd., Shanghai, China) was used for cell viability detection. 

Analogous to a previous study [25], the discharge of LDH in the culture medium was detected according to a LDH Cytotoxicity Assay Kit (C0017, Beyotime Biotechnology, Shanghai, China). 

### 2.5. Nitric Oxide (NO) Assay

Due to its extreme instability, NO is quickly and proportionally metabolized to nitrite. Therefore, nitrite has been extensively used as an indicator of NO [26]. BV2 cells (1.5 × 10^5^ per well) were cultured in 6-well plates overnight. Then BV2 cells were subjected to the 3 h incubation of VB (50 μM and 100 μM) followed by 24 h stimulation with 1 μg/mL LPS. For the co-culture, N2a cells (1.5 × 10^5^ per well) grown in 6-well plates were subjected to BV2 conditional medium (2 mL) for 24 h. The harvested cells were lysed by cell lysis buffer (P0013, Beyotime, Shanghai, China), and the supernatant was collected after centrifugation at 1.2 × 10^4^ rpm for 5 min. Following the instructions of the manufacturer (S0021S, Beyotime, Shanghai, China), the level of nitrite was analyzed at 540 nm. 

### 2.6. Transmission Electron Microscopy (TEM)

Pretreatment of VB (50 μM and 100 μM) was given to BV2 cells for 3 h followed by 24 h stimulation with 1 μg/mL LPS, and then fixed with 2.5% glutaraldehyde (R20513, Shanghai YuanYe, Shanghai, China) for 5 min. The gently collected cells were fixed with new 2.5% glutaraldehyde solution at 4 °C for 8 h. The ultrastructure of the BV2 cells was ascertained through TEM (H-7650, HITACHI, Tokyo, Japan).

### 2.7. Immunohistochemistry and Immunofluorescence

Immunohistochemistry and immunofluorescence for brain samples were performed, with some modifications mentioned from our previous study [23]. Briefly, the brain tissue samples were inlayed in paraffin, treated with gradient ethanol, sealed with 3% H_2_O_2_, and incubated with primary antibodies (Supplementary Material Table S1) firstly and then secondary antibodies (Table S1). The representative photos of samples were acquired under an optical microscope (BX51, Olympus, Tokyo, Japan) and fluorescence microscope (BX53, Olympus, Tokyo, Japan), respectively.

Furthermore, BV2 cells (8 × 10^4^ cells per well) were subjected to the 3 h pretreatment with VB (50 μM and 100 μM) and then hatched with 1 μg/mL LPS for 24 h. Similarly, 3 h pretreatment of VB (25 μM and 50 μM) was given to N2a cells (1 × 10^5^ cells per well) before stimulating with 5 μM Aβ_1-42_ for another 24 h. NF-κB-p65 translocation was analyzed using the NF-κB activation-nuclear translocation assay kit (SN368, Beyotime, Shanghai, China) as per the instructions of the manufacturer. Fluorescence images were received using a laser scanning confocal microscope (LSM710, Carl Zeiss Meditec, Jena, Germany). 

### 2.8. Enzyme-Linked Immunosorbent Assay (ELISA)

Brain tissues were ground to collect proteins. The cerebral levels of brain-derived neurotrophic factor (BDNF) (RK00433), TGF-β (RK00057), IL-1β (RK00006), IL-6 (RK00008), and monocyte chemoattractant protein-1 (MCP-1) (RK00381) were detected with commercial kits (ABclonal, Wuhan, China), following the instructions of the manufacturer. 

Cultured BV2 cells were subjected to the 3 h incubation of VB (50 μM and 100 μM) and then 24 h stimulation of 1 μg/mL LPS. IL-1β (EK0394) as well as IL-6 (EK0411) levels in the cultured medium were detected with commercial kits (Boster, Wuhan, China) accordingly.

### 2.9. Western Blot

The pretreatment of VB (50 μM and 100 μM) was offered to BV2 cells for 3 h before 24 h stimulation of 1 μg/mL LPS. The pretreatment of VB (25 μM and 50 μM) was given to N2a cells for 3 h before 24 h stimulation of 5 μM Aβ_1-42_. Brain tissue, BV2 cells, and N2a cells were lysed to collect proteins, which were normalized using a BCA Protein Assay Kit (23225, Thermo Scientific, Waltham, MA, USA), separated by a one-step PAGE gel fast preparation kit (PG212 and PG213, Epizyme Biotech, Shanghai, China), and shifted onto polyvinylidene fluoride (PVDF) membranes (88585, Thermo, Waltham, MA, USA) before incubation of antibodies (Table S1). An automatic chemiluminescence image analysis system (Tanon 5200, Tanon Science & Technology Co., Ltd., Shanghai, China) was introduced to take the photos of protein bands, and the protein concentration was quantified using ImageJ 6.0 software (National Institutes of Health, Bethesda, MD, USA). 

### 2.10. Statistical Analysis

Data are presented in the form of mean ± standard error of mean (SEM). Tukey’s post hoc test was performed based on results of one-way analysis of variance (ANOVA) by BONC DSS Statistics 25 software (Business Intelligence of Oriental Nations Co., Ltd., Beijing, China) to analyze the data. GraphPad Prism 9 software (GraphPad Software Inc., San Diego, CA, USA) was utilized to generate graphs, and *p* < 0.05 was defined as statistically significant.

## 3. Results

### 3.1. Neuroprotection of VB Links Closely with Microglia and Astrocyte Activation

**Figure 1:**
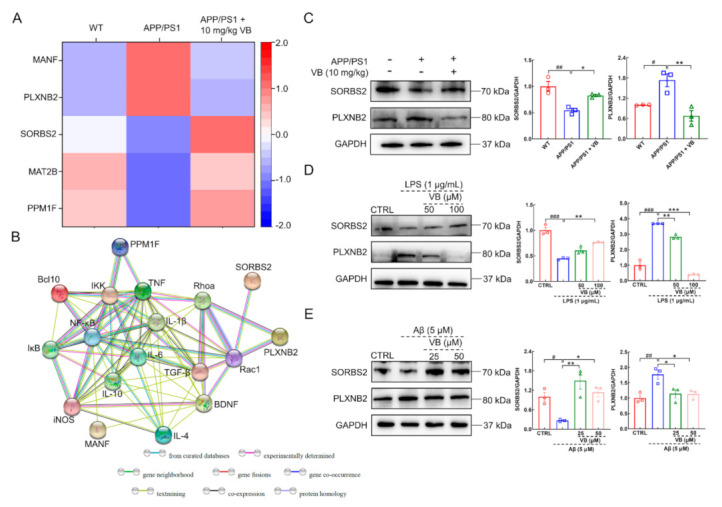
The neuroprotection of VB correlates to its anti-inflammatory properties. (**A**) Heat map of 5 proteins in APP/PS1 mice. Red means high-abundant proteins, while blue means low-abundant proteins. (**B**) The significantly different proteins, including SORBS2 and PLXNB2, interacted closely with inflammation-related factors via the protein–protein interaction network analysis. The production of SORBS2 and PLXNB2 in (**C**) APP/PS1 mice, (**D**) LPS-induced BV2 cells, and (**E**) Aβ_1-42_-stimulated N2a cells was detected through Western blot, quantified through densitometry, and expressed as the fold of control group (*n* = 3). Results are shown as means ± SEM, ^#^ *p* < 0.05, ^##^ *p* < 0.01 vs. WT mice, * *p* < 0.05, ** *p* < 0.01 vs. APP/PS1 mice for (**C**). ^###^ *p* < 0.001 vs. CTRL BV2 cells, ** *p* < 0.01, *** *p* < 0.001 vs. LPS-induced BV2 cells for (**D**). ^#^ *p* < 0.05, ^##^ *p* < 0.01 vs. CTRL N2a cells, * *p* < 0.05, ** *p* < 0.01 vs. Aβ_1-42_-stimulated N2a cells for (**E**).

In view of the results of the proteomics analysis, five proteins associated with inflammation were identified (Figure 1A,B; Table S2) in brains of APP/PS1 mice. The level of Sorbin and SH3 domain-containing protein 2 (SORBS2) lowered in the brains of APP/PS1 mice. SORBS2 suppressed the expression of IL-6 and partially blocked the NF-κB-p65 pathway after Toll-like receptor stimulation [27]. The level of Plexin-B2 (PLXNB2) was enhanced in the brains of APP/PS1 mice. PLXNB2 could regulate NF-κB-p65 and inflammasomes, thus aggravating inflammatory reaction in psoriasis [28]. Accordingly, the changes in the expression of SORBS2 and PLXNB2 were related to ILs and NF-κB-p65 signaling. 

VB elevated the reduced generation of SORBS2 and suppressed the increased generation of PLXNB2 in the brains of APP/PS1 mice (*p* < 0.05) (Figure 1C), LPS-exposed BV2 cells (*p* < 0.01) (Figure 1D), and Aβ_1-42_-stimulated N2a cells (*p* < 0.05) (Figure 1E). Results mentioned above imply the neuroprotection of VB in AD is connected with its anti-inflammatory properties. 

**Figure 2:**
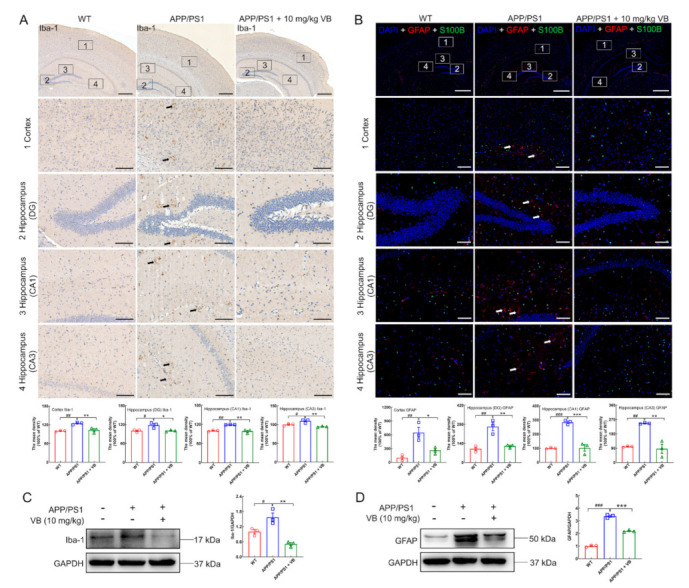
VB prevents microglia and astrocyte against activation in brains of APP/PS1 mice. (**A**) Representative photos and semi-quantitative data of Iba-1 in the (1) cortex, (2) hippocampus DG, (3) hippocampus CA1, and (4) hippocampus CA3 (*n* = 3). The black arrows indicate Iba-1-positive microglia. Scale bar = 400 μm for 50× magnification, and scale bar = 100 μm for 200× magnification. (**B**) Representative images and semi-quantitative data of GFAP and/or S100B in the (1) cortex, (2) hippocampus DG, (3) hippocampus CA1, and (4) hippocampus CA3 (*n* = 3). Red represents GFAP-positive astrocytes, while green represents S100B-positive astrocytes. The white arrows represent GFAP-positive astrocytes. Scale bar = 800 μm for 50× magnification, and scale bar = 200 μm for 200× magnification. The production of (**C**) Iba-1 and (**D**) GFAP were determined through Western blot, quantified via densitometry, and presented as the fold of WT group (*n* = 3). Results were presented as means ± SEM, ^#^ *p* < 0.05, ^##^ *p* < 0.01 and ^###^ *p* < 0.001 vs. WT mice, * *p* < 0.05, ** *p* < 0.01 and *** *p* < 0.001 vs. APP/PS1 mice.

Microglia and astrocytes, the major glial cells in the CNS, are responsible for the initiation and development of neuroinflammation [8]. Accordingly, contrasted with APP/PS1 mice, the generation of ionized calcium-binding adapter molecule-1 (Iba-1), a classic marker of microglial activation [29], was strongly suppressed by VB administration in cortex (*p* < 0.01), hippocampus (DG) (*p* < 0.05), hippocampus (CA1) (*p* < 0.01), and hippocampus (CA3) (*p* < 0.01) (Figure 2A). Glial fibrillary acidic protein (GFAP) functions as a marker of activated astrocytes [29], which was lessened by VB in cortex (*p* < 0.05) (Figure 2B and Figure S2), hippocampus (DG) (*p* < 0.01) (Figure 2B), hippocampus (CA1) (*p* < 0.001) (Figure 2B), and hippocampus (CA3) (*p* < 0.01) (Figure 2B). However, S100 calcium binding protein B (S100B), another astrocyte marker [30], showed no significant discrepancy among all experimental groups (Figure 2B and Figure S3). These results were further confirmed by Western blot, which demonstrated that VB suppressed the production of Iba-1 (*p* < 0.01) (Figure 2C) and GFAP (*p* < 0.001) (Figure 2D) in the brain samples of APP/PS1 mice.

### 3.2. VB Mediates Anti-Neuroinflammatory Properties through Modulation of NF-κB-p65 Signaling in APP/PS1 Mice

**Figure 3:**
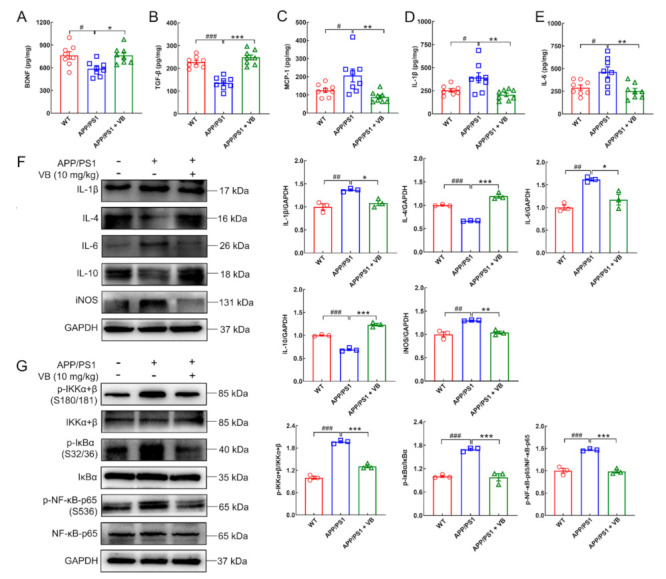
VB regulates neuroinflammation in brains of APP/PS1 mice through NF-κB-p65 signaling. In the brain of APP/PS1 mice, VB regulated the production of (**A**) BDNF, (**B**) TGF-β, (**C**) MCP-1, (**D**) IL-1β, and (**E**) IL-6 as determined through ELISA (*n* = 8); lowered the production (**F**) of IL-1β, IL-6 and iNOS, enhanced that of anti-inflammatory factors including IL-4 and IL-10 (*n* = 3), and blocked (**G**) the activation of the NF-κB-p65 pathway (*n* = 3). Data from Western blot were quantified via densitometry and expressed as the fold of WT group. Results were presented as means ± SEM, ^#^ *p* < 0.05, ^##^ *p* < 0.01 and ^###^ *p* < 0.001 vs. WT mice, * *p* < 0.05, ** *p* < 0.01 and *** *p* < 0.001 vs. APP/PS1 mice.

BDNF is a neurotrophic factor involved in synaptic plasticity and neurogenesis [31]. Based on the prior conclusion, BDNF works as a bridge that links neurogenesis and inflammation [32]. According to a report, BDNF is inhibited in the hippocampus of type-1 diabetes mice, after NF-κB was activated [33]. In this study, VB significantly upregulated BDNF expression (*p* < 0.05) (Figure 3A), thus confirming the neuroprotective role of VB in APP/PS1 mice. 

Additionally, reduced expression of TGF-β (*p* < 0.001) (Figure 3B), overexpression of MCP-1 (an inflammatory-activation chemokine involved in recruiting monocytes and promoting inflammation [34]) (*p* < 0.05) (Figure 3C), and pro-inflammatory cytokines IL-1β (*p* < 0.05) (Figure 3D) and IL-6 (*p* < 0.05) (Figure 3E) were noted in the brains of APP/PS1 mice. VB treatment reversed production of all (*p* < 0.01) (Figure 3B–E).

These results were further evidenced through Western blot. In APP/PS1 mice, VB significantly lessened the generation of IL-1β (*p* < 0.05), IL-6 (*p* < 0.05), and inducible nitric oxide synthase (iNOS) (an enzyme engaged in the generation of NO [35]) (*p* < 0.01) and boosted the production of anti-inflammatory factors, including IL-4 (*p* < 0.001) and IL-10 (*p* < 0.001) (Figure 3F). Moreover, VB effectively inhibited the phosphorylated levels of I kappa B kinase (IKK)α+β, inhibitor of κBα (IκBα), as well as NF-κB-p65 in the brains of APP/PS1 mice (*p* < 0.001) (Figure 3G). Consequently, VB exerts anti-neuroinflammatory properties via modulating NF-κB-p65 pathway in brains of APP/PS1 mice.

### 3.3. VB Exerts Anti-Inflammatory Properties in LPS-Induced BV2 Cells by Regulating NF-κB-p65 Signaling 

**Figure 4:**
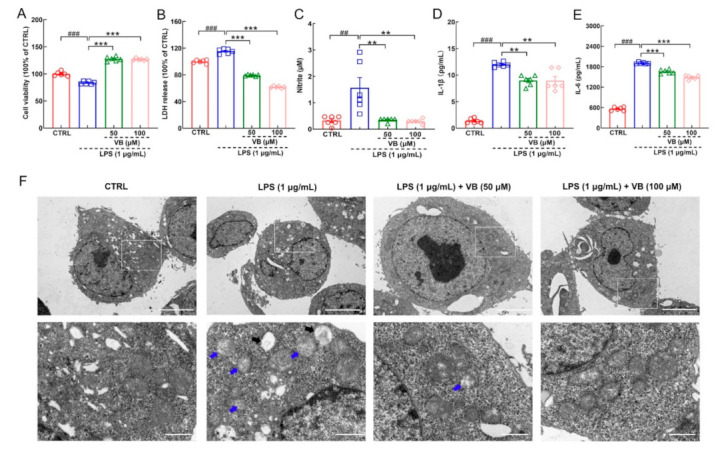
VB possesses anti-inflammatory properties in LPS-induced BV2 cells. VB (**A**) enhanced the viability and suppressed the production of (**B**) LDH, (**C**) NO, (**D**) IL-1β, and (**E**) IL-6 (*n* = 6) in the LPS-induced BV2 cells. (**F**) Representative images of LPS-induced BV2 cells in TEM analysis (*n* = 3). Scale bar = 5 μm for 1500× magnification; scale bar = 1 μm for 6000× magnification. The blue arrows represent the dissolving mitochondria. The black arrows represent cavities after mitochondria dissolution. Results were presented as means ± SEM, ^##^ *p* < 0.01 and ^###^ *p* < 0.001 vs. CTRL BV2 cells, ** *p* < 0.01 and *** *p* < 0.001 vs. LPS-induced BV2 cells.

VB improved the viability of LPS-exposed BV2 cells (*p* < 0.001) (Figure 4A) and lowered the discharge of LDH (*p* < 0.001) (Figure 4B), indicating its protective effect against LPS-induced cell damage. VB suppressed the expression of NO (*p* < 0.01) (Figure 4C), IL-1β (*p* < 0.01) (Figure 4D), as well as IL-6 (*p* < 0.001) (Figure 4E). According to the TEM analysis, VB attenuated the dissolution of mitochondria (Figure 4F) and improved mitochondrial dysfunction, thereby alleviating inflammation [36]. Accordingly, VB effectively suppressed inflammation in the LPS-induced BV2 cells. 

**Figure 5:**
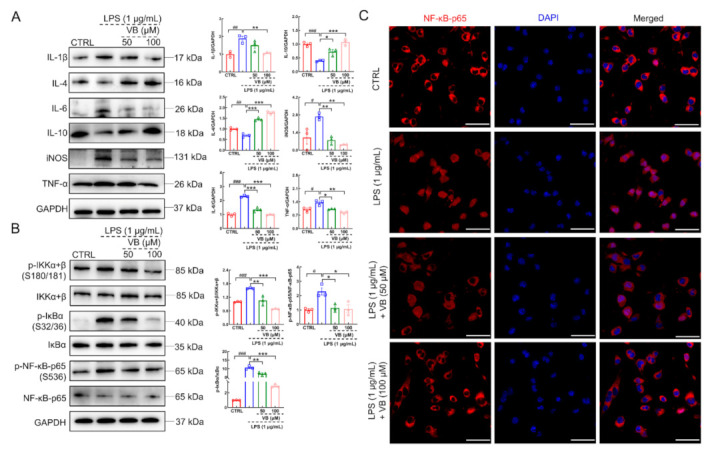
VB inhibits NF-κB-p65 signaling to show anti-inflammatory properties in LPS-induced BV2 cells. In BV2 cells induced by LPS, (**A**) VB suppressed the expression levels of pro-inflammatory factors, enhanced those of anti-inflammatory factors, and (**B**) repressed the phosphorylation of IKKα+β, IκBα, and NF-κB-p65. Data from Western blot were quantified via densitometry and expressed as the fold of CTRL (*n* = 3). (**C**) VB lowered the tendency of NF-κB-p65 translocation towards nucleus in BV2 cells induced by LPS (*n* = 3). Scale bar = 50 μm for 400× magnification. Results were presented as means ± SEM, ^#^ *p* < 0.05, ^##^ *p* < 0.01 and ^###^ *p* < 0.001 vs. CTRL BV2 cells, * *p* < 0.05, ** *p* < 0.01 and *** *p* < 0.001 vs. LPS-induced BV2 cells.

Compared to LPS-damaged BV2 cells, VB significantly downregulated the production of IL-1β (*p* < 0.01), IL-6 (*p* < 0.001), iNOS (*p* < 0.01), and TNF-α (*p* < 0.05) and upregulated the expression of IL-4 (*p* < 0.001) and IL-10 (*p* < 0.05) (Figure 5A). This observation further verified the repression of VB on inflammation. The enhanced phosphorylated IKKα+β, IκBα, and NF-κB-p65 caused by LPS stimulation in BV2 cells were significantly restrained by VB (*p* < 0.05) (Figure 5B), suggesting that NF-κB signaling plays a crucial role in the anti-inflammation effect of VB. In LPS-induced BV2 cells, NF-κB-p65 has the tendency of translocation towards nucleus to support the transcription of downstream pro-inflammatory factors such as IL-1β [14]. However, VB prevented NF-κB-p65 from translocating towards nucleus in LPS-treated BV2 cells dose-dependently (Figure 5C). 

### 3.4. VB Exerts Neuroprotection in Aβ_1-42_-Stimulated N2a Cells by Inhibiting Neuroinflammation

**Figure 6:**
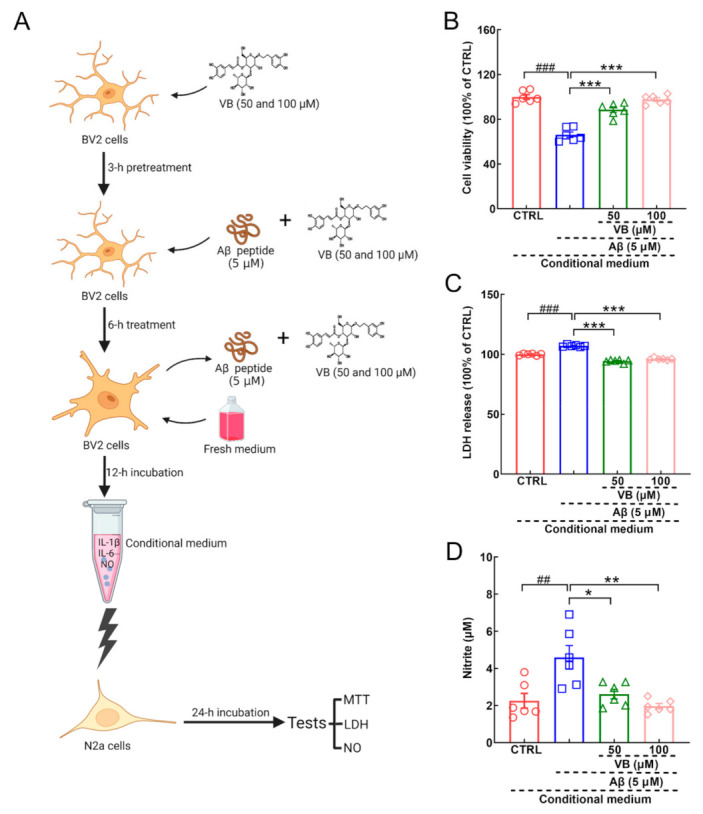
VB shows neuroprotective effect on N2a cells cultured with conditional medium from Aβ_1-42_-treated BV2 cells. (**A**) The schematic diagram of co-culturing experiments. Created with Biorender.com. For N2a cells cultured in conditional medium, VB (**B**) improved the viability and suppressed the production of (**C**) LDH and (**D**) NO (*n* = 6). Results were shown as means ± SEM, ^##^ *p* < 0.01, ^###^ *p* < 0.001 vs. N2a cells exposed in conditional medium from CTRL BV2 cells, * *p* < 0.05, ** *p* < 0.01 and *** *p* < 0.001 vs. N2a cells exposed in conditional medium from Aβ_1-42_-stimulated BV2 cells.

During the development of AD, microglia sense extracellular Aβ plaques and release cytokines and chemokines into the microenvironment, which finally affect the function of neurons [13]. To mimic this pathological condition, cellular co-culture was performed to assess the link between neuroprotection and neuroinflammation (Figure 6A). Lessened cell viability (*p* < 0.001) (Figure 6B), enhanced levels of LDH in the culture medium (*p* < 0.001) (Figure 6C), and upregulated levels of NO (*p* < 0.01) (Figure 6D) were found in N2a cells exposed to the conditional medium from Aβ_1-42_-treated BV2 cells. VB incubation reversed all these changes to protect N2a cells against stimuli from pro-inflammatory factors (*p* < 0.05) (Figure 6B–D).

**Figure 7:**
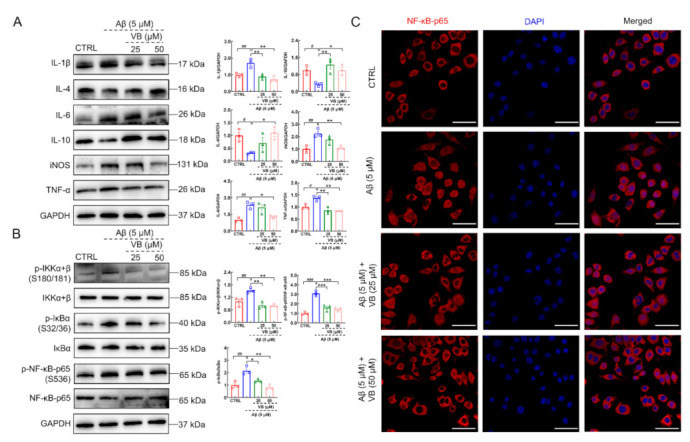
VB inhibits neuroinflammation in the Aβ_1-42_-stimulated N2a cells through regulating the NF-κB-p65 signaling. (**A**) VB restrained the expression levels of IL-1β, IL-6, iNOS, and TNF-α and upregulated those of IL-4 and IL-10 in Aβ_1-42_-stimulated N2a cells (*n* = 3). (**B**) VB restrained the activation of the NF-κB-p65 signaling in N2a cells stimulated by Aβ_1-42_ (*n* = 3). Data from Western blot were quantified via densitometry and expressed as the fold of CTRL. (**C**) VB lowered the tendency of NF-κB-p65 translocation towards nucleus in N2a cells stimulated by Aβ_1-42_ (*n* = 3). Scale bar = 50 μm for 400× magnification. Results were presented as means ± SEM, ^#^ *p* < 0.05, ^##^ *p* < 0.01 and ^###^ *p* < 0.001 vs. CTRL N2a cells, * *p* < 0.05, ** *p* < 0.01 and *** *p* < 0.001 vs. Aβ_1-42_-stimulated N2a cells.

VB dose-dependently improved the viability of Aβ_1-42_-stimulated N2a cells, further confirming the neuroprotection of VB against AD (*p* < 0.001) (Figure S4). In Aβ_1-42_-exposed N2a cells, VB significantly downregulated the levels of IL-1β (*p* < 0.01), IL-6 (*p* < 0.05), iNOS (*p* < 0.01), and TNF-α (*p* < 0.01) and upregulated the expression of IL-4 (*p* < 0.05) and IL-10 (*p* < 0.05) (Figure 7A). Compared to Aβ_1-42_-exposed N2a cells, the elevated phosphorylated IKKα+β, IκBα, and NF-κB-p65 was reversed by VB treatment (*p* < 0.05) (Figure 7B), which also prevented NF-κB-p65 from translocating towards nucleus (Figure 7C).

## 4. Discussion

Earlier, our team preliminarily demonstrated that VB significantly promoted the elimination of Aβ plaques and NFTs [23]; however, the exact mechanism by which VB exerts neuroprotection in AD remains unclear. In this study, we explored the anti-inflammatory role of VB in APP/PS1 mice based on proteomics analysis. VB prevented microglia and astrocytes against activation and restrained the expression levels of iNOS, NO, and other pro-inflammatory cytokines and the activation of NF-κB-p65 signaling-related proteins. These results were further confirmed in LPS-induced BV2 and Aβ_1-42_-stimulated N2a cells. Therefore, VB plays a neuroprotective role in AD, as it inhibits neuroinflammation by blocking the NF-κB-p65 pathway.

Through positron emission tomography, researchers have evidenced neuroinflammation takes place in the early stage of AD [37,38]. GV-971 has been confirmed to reverse cognition dysfunction in mild-to-moderate AD by regulating neuroinflammation [18]. Therefore, neuroinflammation is a vital aspect to focus on during the prevention and treatment of AD. In this study, based on the results of proteomic analysis, the expressions of PLXNB2 and SORBS2 were significantly discrepant among all experimental groups, which are closely associated with inflammation. PLXNB2 could promote inflammatory response both in allergic airway inflammation and psoriasis [39,40]. In the CNS, PLXNB2 released by astrocytes caused inflammation in multiple sclerosis and experimental autoimmune encephalomyelitis by connecting with CD100 (the ligand of PLXNB2) in microglia [41]. Additionally, the downregulated SORBS2 was observed in the heart tissues of LPS-treated C57BL/6 mice [42]. The silence of SORBS2 in mouse embryonic fibroblasts led to the higher expression of *IL-6* RNA [27]. Accordingly, VB regulated the levels of PLXNB2 and SORBS2 in brains of APP/PS1 mice, LPS-induced BV2 cells, and Aβ_1-42_-exposed N2a cells, which implied the neuroprotection of VB was related to the regulation of inflammation.

Neuroinflammation is characterized by the activation of astrocytes and microglia. There are two extreme types of activated microglia: the pro-inflammatory M1 subtype (classically activated) and the anti-inflammatory M2 subtype (alternatively activated) [43]. In our study, VB lessened the production of M1 markers including iNOS, IL-1β, IL-6, and TNF-α [44]. This observation suggests that VB mitigates the M1 polarization of microglia. Furthermore, VB upregulated the levels of anti-inflammatory cytokines such as IL-4, IL-10 as well as TGF-β in the APP/PS1 mice. Interestingly, in the CNS, TGF-β discharged by mesenchymal stromal cells played an critical role in transforming the M1 microglia to M2 subtype and attenuating NF-κB signaling [45]. TGF-β produced by phagocytizing microglia could block the production of autocrine TNF-α and repress oxidative stress, thus supporting the survival of phagocytizing microglia [46]. These studies imply that TGF-β could regulate the function of microglia towards that of M2 subtype, thus dissolving neuroinflammation. Consequently, VB regulates the polarization of microglia.

During the early onset of AD, scavenger receptors (SRs) are abundant on the membranes of activated microglia and are involved in the engulfment of Aβ deposits [47]. However, persistent stimulation caused by Aβ plaques leads to chronic neuroinflammation and increased release of pro-inflammatory factors, such as TNF-α, IL-1β, and IL-6 from the activated microglia, which in turn inhibits the expression levels of SRs. This promotes the aggregation of Aβ plaques that is followed by exacerbation of neuroinflammation and AD pathology [43,48,49]. Pro-inflammatory factors secreted by microglia activate astrocytes to produce more pro-inflammatory mediators, causing an amplification of neuroinflammation [50]. Consistently, the reactive microglia and astrocytes in the brains of APP/PS1 mice were significantly repressed by VB, thus confirming that VB exerts neuroprotection via neuroinflammation prevention.

Apart from extracellular Aβ plaques and intracellular NFTs, deficiency in neurons is also noted in patients with AD [51]. BDNF is responsible for neuronal survival and synaptic plasticity, which helps in alleviating cognitive impairment [52]. A higher level of BDNF was observed in the APP/PS1 mice after VB administration, confirming its anti-AD properties. Glia-neuron interactions are crucial for AD pathology. The pro-inflammatory cytokines released by microglia not only cause direct toxicity to neurons but also aggregate the formation of Aβ plaques, which forms a vicious cycle during AD pathology associated with neuroinflammation [38]. Our cellular co-culture experiment was consistent with this conclusion. In Aβ_1-42_-stimulated N2a cells, VB effectively dissolved inflammatory response as evidenced by the lower levels of pro-inflammatory mediators and higher expression of anti-inflammatory ones. The activation of microglia and astrocytes has been reported to be closely linked with Aβ plaque formation and tau pathology, thereby leading to the occurrence of AD-like symptoms such as memory decline and learning disorders [53]. Nevertheless, in our previous study, VB improved AD-related symptoms in APP/PS1 mice [23]. These results confirm the important role of the anti-neuroinflammatory effect of VB in AD.

According to previous studies, NF-κB, a member of the transcription factor Rel family, regulates neuroinflammation [54]. Once stimuli such as Aβ plaques and LPS are recognized by microglia, IKKα+β, an inhibitor of κB kinase, is phosphorylated. This is followed by the phosphorylation of IκBα at serine 32 and 36 positions [14,55]. IκBα originally functions to prevent NF-κB from translocation towards nucleus. After the phosphorylated IκBα dissociating from p50/p65, the NF-κB heterodimer successfully translocate towards nucleus for further transcription of pro-inflammatory factors [14]. Similar changes were observed in VB-treated APP/PS1 mice, LPS-exposed BV2 cells, and Aβ_1-42_-stimulated N2a cells. The anti-neuroinflammatory effect of VB suppresses the activation of NF-κB-p65 signaling.

We noted that this study had some limitations. Since there are many regulators that are implicated in NF-κB signaling [56], the effect of VB exposure on upstream regulators of NF-κB signaling needs to be explored further, both in glial cells and neurons. Additionally, researchers have evidenced that PLXNB2-CD100 engaged in the regulation of neuroinflammation via the microglia-astrocyte communication [41]. Our study has also verified the essential role of PLXNB2 in the VB-mediated amelioration of neuroinflammation. It may be interesting to further explore whether VB engages in the regulation of microglia-astrocyte communication and whether PLXNB2-CD100 is also involved during this process.

## 5. Conclusions

Contrasted with APP/PS1 mice, VB significantly prevented microglia and astrocyte against activation by regulating the NF-κB-p65 pathway. VB suppressed the production of pro-inflammatory mediators and upregulated those of anti-inflammatory ones by blocking NF-κB-p65 from translocating towards nucleus in LPS-treated BV2 cells. VB protects N2a cells against stimulation of conditional medium from Aβ_1-42_-treated BV2 cells and also plays a neuroprotective role by inhibiting neuroinflammation in Aβ_1-42_-exposed N2a cells. Consequently, VB-mediated anti-AD effects are at least partly related to its anti-neuroinflammatory properties, which contributes to its neuroprotective mechanism and provided more experimental evidence for its application as a promising treatment for AD.

## Data Availability

The data sets and materials supporting the conclusions of this study are included within the article and its supplementary information file.

## Supplementary Materials

The following supporting information can be downloaded at: https://www.mdpi.com/article/10.3390/nu14071417/s1. Table S1: List of antibodies introduced in immunohistochemistry, immunofluorescence, and Western blot. Table S2: Proteins with significantly discrepant expression levels in proteomics. Figure S1: The chemical structure of VB (CAS: 61276-17-3). Figure S2: (A) VB inhibited the expression of GFAP (black arrow) in the cortex of APP/PS1 mice through immunohistochemistry (*n* = 3). Scale bar = 400 μm for 50× magnification, and scale bar = 100 μm for 200× magnification. (B) The quantitative analysis of GFAP in WT, APP/PS1, and VB-treated APP/PS1 mice (*n* = 3). ^###^ *p* < 0.001 vs. WT mice; *** *p* < 0.001 vs. APP/PS1 mice. Figure S3: The expression of S100B showed no significant discrepancy in the (A) cortex, (B) hippocampus (DG), (C) hippocampus (CA1), or (D) hippocampus (CA3) among all experimental groups (*n* = 3). Figure S4: VB dose-dependently improved the viability of Aβ_1-42_-induced N2a cells (*n* = 6). ^###^ *p* < 0.001 vs. CTRL N2a cells; *** *p* < 0.001 vs. Aβ_1-42_-stimulated N2a cells.
