# Supplementary material for: The Neuroprotection of Verbascoside in Alzheimer’s Disease Mediated through Mitigation of Neuroinflammation via Blocking NF-κB-p65 Signaling
Source: Nutrients. 2022 Mar 29;14(7):1417. doi: 10.3390/nu14071417 (PMC9003273; doi:10.3390/nu14071417)
Supplement: Supplementary file 1 [file nutrients-14-01417-s001.zip › nutrients-1631154 Supplementary data.pdf]

Table S1. List of antibodies introduced in immunohistochemistry, immunofluorescence, and western blot

Antibody	Molecular weight	Catalog number	Dilution	Application	Company	Area
GFAP	50 kDa	PB9082	1:400	Immunohistochemistry	Boster	Wuhan, China
Iba-1	17 kDa	10904-1-AP	1:400	Immunohistochemistry	Proteintech	Wuhan, China
GFAP	49 kDa	GB12096	1:500	Immunofluorescence	Servicebio	Wuhan, China
S100B	11 kDa	GB11359	1:500	Immunofluorescence	Servicebio	Wuhan, China
SORBS2	70 kDa	24643-1-AP	1:1000	Western blot	Proteintech	Wuhan, China
PLXNB2	80 kDa	A10069	1:1000	Western blot	ABclonal	Wuhan, China
GFAP	50 kDa	80788S	1:1000	Western blot	Cell Signaling Technology	Beverly, MA, USA
Iba-1	17 kDa	A19776	1:1000	Western blot	ABclonal	Wuhan, China
IL-1β	17 kDa	A1112	1:1000	Western blot	ABclonal	Wuhan, China
IL-4	16 kDa	A4988	1:1000	Western blot	ABclonal	Wuhan, China
IL-6	26 kDa	A0286	1:1000	Western blot	ABclonal	Wuhan, China
IL-10	18 kDa	A2171	1:1000	Western blot	ABclonal	Wuhan, China
iNOS	131 kDa	A3200	1:750	Western blot	ABclonal	Wuhan, China
TNF-α	26 kDa	17590-1-AP	1:1000	Western blot	Proteintech	Wuhan, China
p-IKKα+β (S180/181)	85 kDa	AF3013	1:1000	Western blot	Affinity	Cincinnati, OH, USA

IKKα+β	85 kDa	ab178870	1:1000	Western blot	Abcam	Cambridge, MA, USA
p-IκBα (S32/36)	40 kDa	ab12135	1:500	Western blot	Abcam	Cambridge, MA, USA
IκBα	35 kDa	ab32518	1:4000	Western blot	Abcam	Cambridge, MA, USA
p-NF-κB-p65 (S536)	65 kDa	AF2006	1:1000	Western blot	Affinity	Cincinnati, OH, USA
NF-κB-p65	65 kDa	ab16502	1:4000	Western blot	Abcam	Cambridge, MA, USA
GAPDH	37 kDa	E-AB-20032	1:4000	Western blot	Elabscience	Wuhan, China
goat anti-mouse		E-AB-1001	1:4000	Western blot	Elabscience	Wuhan, China
goat anti- rabbit		E-AB-1003	1:4000	Western blot	Elabscience	Wuhan, China
Goat anti-rabbit		BA1003	1:150	Immunohistochemistry	Boster	Wuhan, China
Cy3 conjugated goat anti-mouse IgG (H+L)		GB21301	1:300	Immunofluorescence	Servicebio	Wuhan, China
Alexa Fluor® 488-conjugated goat anti-rabbit IgG (H+L)		GB25303	1:400	Immunofluorescence	Servicebio	Wuhan, China

Table S2. Proteins with significantly discrepant expression levels in proteomics

Number	Protein. names	Unique. peptides	fc. WT-APP/PS1	fc. APP/PS1+VB-APP/PS1
Upregulated proteins by VB (Number: 3)				
1	PPM1F	3	4.843248	5.910574
2	MAT2B	5	7.203061	6.646857
3	SORBS2	8	4.168305	7.455264
Downregulated proteins by VB (Number: 2)				
1	PLXNB2	6	0.245348	0.260457
2	MANF	4	0.149661	0.228425

fc. WT-APP/PS1: the ratio of protein between WT mice and vehicle-treated APP/PS1 mice;

fc. APP/PS1+VB-APP/PS1: the ratio of protein between VB-treated APP/PS1 mice and vehicle-treated APP/PS1 mice.

Figure Lists

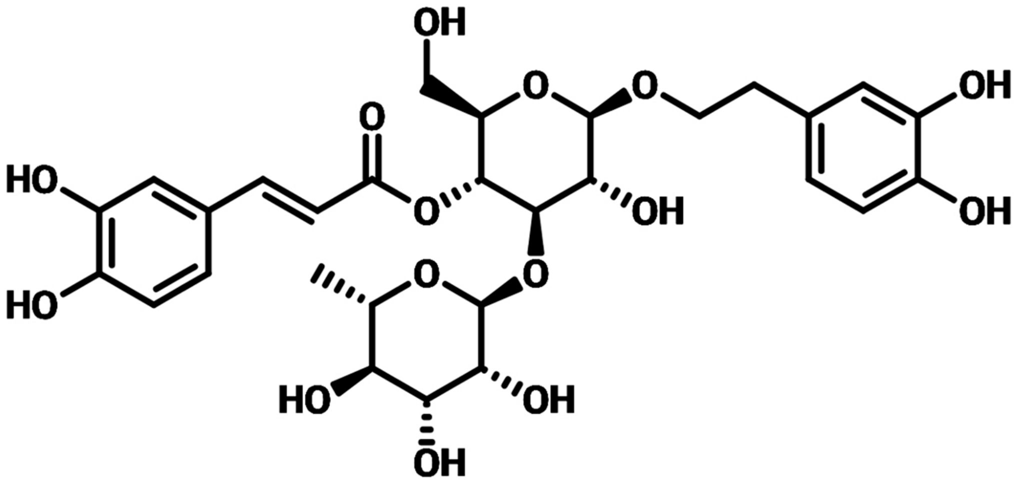

Figure S1: The chemical structure of VB (CAS: 61276-17-3).

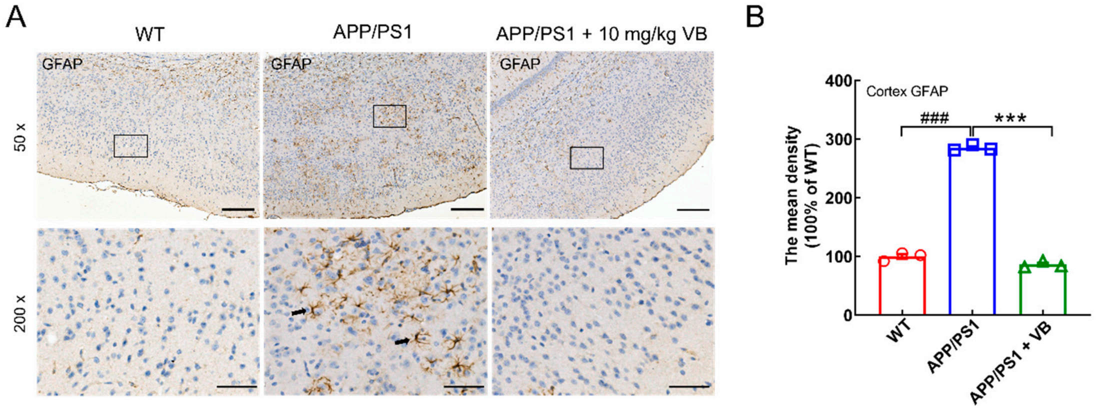

Figure S2: (**A**) VB inhibited the expression of GFAP (black arrow) in the cortex of APP/PS1 mice through immunohistochemistry (n = 3). Scale bar = 400 μm for 50× magnification, and scale bar = 100 μm for 200× magnification. (**B**) The quantitative analysis of GFAP in WT, APP/PS1 and VB-treated APP/PS1 mice (n = 3). $^{###}p < 0.001$ vs. WT mice; $^{***}p < 0.001$ vs. APP/PS1 mice.

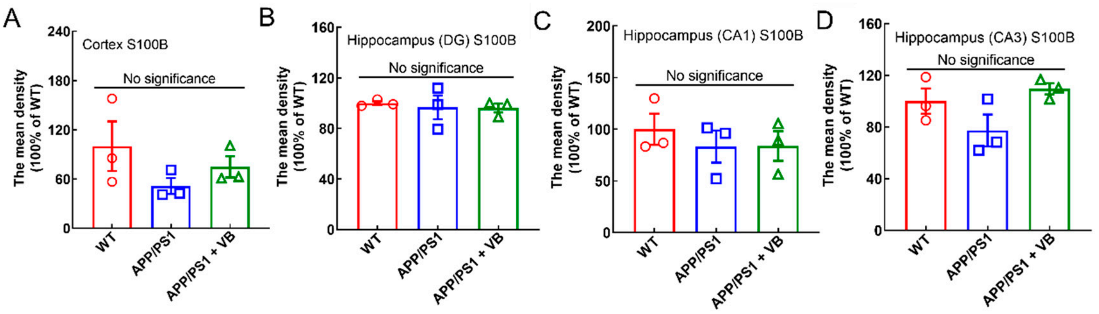

Figure S3: The expression of S100B showed no significant discrepancy in the (**A**) cortex, (**B**) hippocampus (DG), (**C**) hippocampus (CA1), (**D**) hippocampus (CA3) among all experimental groups (n = 3).

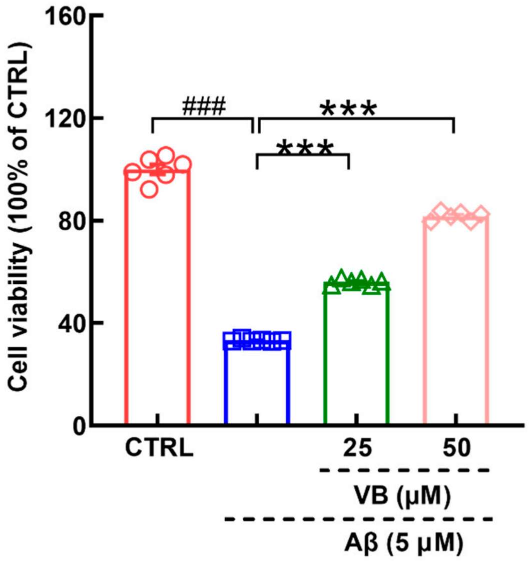

Figure S4: VB dose-dependently improved the viability of $A\beta_{1-42}$-induced N2a cells (n = 6). $^{###}p < 0.001$ vs. CTRL N2a cells; $^{***}p < 0.001$ vs. $A\beta_{1-42}$-stimulated N2a cells.
